# Dr. William Parsons Tritton

**Published:** 1910-08-27

**Authors:** 


### OBITUARY.

The death is reported at Umzinto, Natal, South Africa, of Dr. William Parsons Tritton, M.D. He took his M.R.C.S. and L.S.A. in 1877, and later on he graduated M.D. at Brussels.

